# Molecular and Structure–Properties Comparison of an Anionically Synthesized Diblock Copolymer of the PS-*b*-PI Sequence and Its Hydrogenated or Sulfonated Derivatives

**DOI:** 10.3390/polym13234167

**Published:** 2021-11-28

**Authors:** Nikolaos Politakos, Ioannis Moutsios, Gkreti-Maria Manesi, Konstantinos Artopoiadis, Konstantina Tsitoni, Dimitrios Moschovas, Alexey A. Piryazev, Denis S. Kotlyarskiy, Galder Kortaberria, Dimitri A. Ivanov, Apostolos Avgeropoulos

**Affiliations:** 1 Department of Materials Science Engineering, University Campus-Dourouti, University of Ioannina, 45110 Ioannina, Greece; nikolaos.politakos@ehu.eus (N.P.); imoutsios@uoi.gr (I.M.); gretimanesi@uoi.gr (G.-M.M.); kartopoiades@gmail.com (K.A.); k.tsitoni@uoi.gr (K.T.); dmoschov@uoi.gr (D.M.); 2 POLYMAT and Departamento de Química Aplicada, Facultad de Ciencias Químicas, University of the Basque Country (UPV/EHU), Joxe Mari Korta Zentroa, Tolosa Etorbidea 72, 20018 Donostia-San Sebastián, Spain; 3 Faculty of Chemistry, Lomonosov Moscow State University (MSU), GSP-1, 1-3 Leninskiye Gory, 119991 Moscow, Russia; stunnn@gmail.com (A.A.P.); 9920860@gmail.com (D.S.K.); 4 Institute of Problems of Chemical Physics, Russian Academy of Sciences, Chernogolovka, 142432 Moscow, Russia; 5 Materials+Tecnologies’ Research Group, Chemistry and Environmental Engineering Department, Faculty of Engineering, Gipuzkoa, University of the Basque Country (UPV/EHU), Plaza Europa 1, 20018 Donostia, Spain; galder.cortaberria@ehu.es; 6 Institut de Sciences des Matériaux de Mulhouse—IS2M, CNRS UMR7361, 15 Jean Starcky, 68057 Mulhouse, France

**Keywords:** anionic polymerization, chemical modification reactions, full hydrogenation, partial sulfonation, molecular characterization, thin films, AFM, self-assembly in bulk, SAXS

## Abstract

An approach to obtaining various nanostructures utilizing a well-studied polystyrene-*b*-poly(isoprene) or PS-*b*-PI diblock copolymer system through chemical modification reactions is reported. The complete hydrogenation and partial sulfonation to the susceptible carbon double bonds of the PI segment led to the preparation of [polystyrene-*b*-poly(ethylene-*alt*-propylene)] as well as [polystyrene-*b*-poly(sulfonated isoprene-*co*-isoprene)], respectively. The hydrogenation of the polyisoprene block results in enhanced segmental immiscibility, whereas the relative sulfonation induces an amphiphilic character in the final modified material. The successful synthesis of the pristine diblock copolymer through anionic polymerization and the relative chemical modification reactions were verified using several molecular and structural characterization techniques. The thin film structure–properties relationship was investigated using atomic force microscopy under various conditions such as different solvents and annealing temperatures. Small-angle X-ray scattering was employed to identify the different observed nanostructures and their evolution upon thermal annealing.

## 1. Introduction

A broad class of materials that has attracted significant interest over a wide range of applications in polymer science is diblock copolymers (BCPs). Anionic polymerization has enabled the synthesis of well-defined BCPs exhibiting appealing properties, narrow dispersity and post synthesis modification potential [1,2,3,4,5,6,7]. The ability to combine chemically divergent segments leads to various self-assembled nanodomains such as spheres (BCC), hexagonally close-packed cylinders (HCP), double gyroid (DG) and lamellae (LAM) [8,9,10,11] as well as to uncommon morphologies, rendering the aforementioned systems suitable for nanotechnology [12,13]. These hierarchically ordered nanostructures are strongly dependent on the segment–segment interaction parameter (*χ*) [14], the volume fraction of each block (*φ*_A_, where *φ*_B_ = 1 − *φ*_A_) and the total degree of polymerization (*N*). The covalently connected blocks in diblock copolymers microphase separate due to their thermodynamic immiscibility and the *χΝ* product determines the adopted morphologies above a critical value (equal to 10.5), where the free energy is minimized [9,10,11,12,14]. The use of BCPs in advanced technologies is attributed to their tailored physical and chemical properties originating from their well-defined periodic nanostructures [9].

In order to enhance the properties of BCPs, various post-synthetic modification reactions were adopted on susceptible segments by utilizing different techniques such as hydrogenation, epoxidation, hydrosilylation, sulfonation, chlorination, etc. The synthesis of the most chemically modified materials is impossible using conventional polymerization techniques, as already reported in the literature [7,12]. It is of major importance to mention that the degree of polymerization is not affected by the modification reactions; nevertheless, the interaction parameter, *χ,* between the new segments is significantly altered, leading to differences in the final obtained nanostructures in bulk and/or thin films when compared with the initial pristine copolymer [7]. 

Concerning the hydrogenation of polydienes, and especially that of the poly(isoprene), quite a few routes for modification reaction have been proposed in the literature involving various catalysts, complexes and chemical reagents. Some of them are related to Pd/BaSO_4_ [15], the Ru complex [16,17], the Os complex [18], the Ziegler–Natta type [19], Ni solution [16], Pt/Re [20], Wilkinson [21] catalysts, and p-toluenesulfonyl hydrazide [8,9,22,23,24]. The polystyrene-*b*-poly(isoprene) or PS-*b*-PI diblock copolymer has been extensively studied after hydrogenating the PI segments with one of the aforementioned techniques. It has been proven that non-catalytic hydrogenation using the diimide (N_2_H_2_) procedure may induce adverse effects such as the damaging of polymeric chains, the necessity of high temperatures, and PS saturation [25,26,27,28,29,30,31]. Despite the drawbacks, studies have concluded that by using reducing agents such as p-toluenesulfonyl hydrazide, 100% hydrogenation is feasible under specified conditions, leading to materials with improved properties and/or novel morphologies. On the other hand, procedures involving catalytic systems are of paramount importance mainly in industrial applications. Ziegler-type catalysts are commonly encountered in polymer hydrogenation, leading to high conversion and adequate balance between selectivity and reactivity [25,26,27,28,29,30,31]. 

Regarding the sulfonation modification of the PI block, the most common reactions utilize a complex of sulfur trioxide/1,4 dioxane [23,24,32,33,34] and chlorosulfonyl isocyanate [15]. Sulfur/dioxane complexes are able to act as sulfonation reagents only for PI segments without sulfonating the PS blocks.

Among the most commonly utilized chemical modification techniques is the hydrogenation of polydienes, where the existence of the double bond facilitates the reaction with a number of reagents, inducing desirable physical properties such as thermo-oxidative stability [14,17,19,35] and ultra-violet radiation resistivity [36]. Another method of a post-polymerization chemical modification technique, namely selective block sulfonation, is applied in order to generate materials that present amphiphilic properties in both bulk and thin films providing physical stability to the modified BCPs. It should be mentioned that in the literature such materials have been proposed as micelles, polysoaps, polymeric surfactants, solution modifiers, emulsifiers, wetting agents and foam stabilizers [10,15,32]. Furthermore, polymer electrolyte membranes [37,38,39,40] as well as membranes exhibiting transport/permeation [31] properties were reported using the specific materials. Their use as membranes [41,42] has been expanded to polyelectrolyte brushes as antimicrobial surfaces [22], drug delivery and/or controlled-release systems [22,32]. Post-polymerization modifications have gained remarkable interest in industrial applications as well as in academic research, since the final materials present exquisite properties and are not able to be synthesized using conventional polymerization techniques [43,44,45,46]. 

To the best of our knowledge, surface studies regarding copolymers containing polydiene segments after chemical modification have not yet been reported in the literature, except for a previous work by Politakos et al. [47] involving only hydrogenated linear triblock copolymers from initial ABA-type SIS and SBS samples (B is poly(polybutadiene)-1,4).

**Scheme 1:**
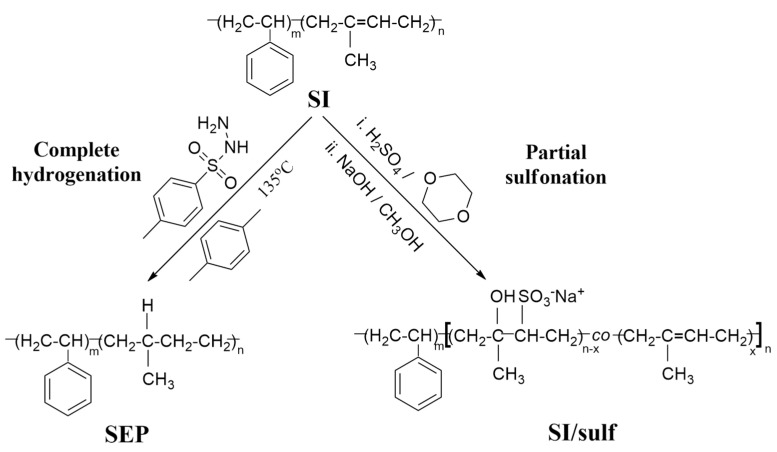
Chemical modification reactions through the complete hydrogenation and partial sulfonation of the initial pristine diblock copolymer (PS-*b*-PI or SI).

In this study, after synthesizing the diblock copolymer of the PS-*b*-PI type via anionic polymerization, two different post-polymerization chemical modifications took place in order to molecularly and morphologically characterize the effect of the hydrogenation and the partial sulfonation of the PI block on the BCP behavior. The well-defined, anionically synthesized diblock copolymer (PS-*b*-PI or SI) was transformed into [polystyrene-*b*-poly(ethylene-*alt*-propylene)] (SEP) after employing the hydrogenation technique, whereas the sulfonation of the PI block resulted in a partially modified amphiphilic material, namely the polystyrene-*b*-poly(sulfonated isoprene-*co*-isoprene) (SI/sulf). The modification reaction scheme is illustrated in Scheme 1.

The polystyrene block remained intact due to the selective reagent attack towards the double bonds of the PI segments, eventually leading to three different chemical structures capable of adopting various morphologies based on the spin casting solution (dilution of the samples in polar and non-polar solvents: THF, toluene, cyclohexane) and annealing conditions used. Specifically, after the successful verification of the chemical modifications through size exclusion chromatography (SEC), nuclear magnetic resonance (NMR) and infrared spectroscopy (IR), all copolymer sequences were morphologically characterized in terms of atomic force microscopy (AFM) in the thin film state. AFM studies were performed for all the copolymer sequences under various parameters such as casting solvent (toluene, tetrahydrofuran and cyclohexane) and different annealing temperatures (room temperature (R.T.), 80 °C, 100 °C and 120 °C). The manipulation of the enthalpic parameters of each sequence holds a significant role on the final adopted morphology, which is strongly affected by the use of selective/non-selective solvents, as well as the enhanced *χ* parameter due to the diminished side-groups (hydrogenation) or the existence of heteroatoms (sulfonation) in the PI blocks. Finally, small-angle X-ray scattering (SAXS) was carried out in order to investigate the morphology of the specific copolymer sequences in bulk, further verifying the temperature effect on the adopted structures.

## 2. Materials and Methods

### 2.1. Materials

The initial diblock copolymer, polystyrene-*b*-polyisoprene (SI), being the precursor for the post-synthetic chemical modification reactions, was synthesized by sequential anionic polymerization under high vacuum techniques. The purification of all reagents involved in the polymerization has already been described in the literature [7,48]. All monomers (styrene, isoprene), solvents (benzene, tetrahydrofuran, methanol, 1,4-dioxane, p-xylene, sulfuric acid, cyclohexane, toluene and deuterated chloroform), initiators (*secondary*-butyl lithium or *sec*-Buli) and reagents such as p-toluenesulfonyl hydrazide were purchased from Sigma-Aldrich (Sigma-Aldrich Co., St Louis, MO, USA). 

The initial SI copolymer was subjected to two different chemical modifications in order to alter, completely or partially, the chemical structure of the PI segment leading either to [polystyrene-*b*-poly(ethylene-alt-propylene)] (SEP) after complete hydrogenation or to polystyrene-*b*-poly(sulfonated isoprene-*co*-isoprene) (SI/sulf) after partial sulfonation. 

The complete hydrogenation of the PI block was achieved using the reducing agent p-toluenesulfonyl hydrazide in p-xylene at 135 °C, as it has already been described in the literature [22]. The exclusive partial sulfonation of the PI segment was realized taking advantage of a sulfur trioxide/1,4-dioxane complex, as already reported elsewhere [33,34].

The analytical synthetic protocol of all diblock copolymer sequences (pristine and chemically modified) is described in the Supporting Information. 

### 2.2. Methods 

The molecular characterization of the initial copolymer and its derivatives was conducted by size exclusion chromatography using a PerkinElmer (Waltham, MA, USA) chromatograph equipped with a binary pump and a refractive index (RI) detector. The eluent used was THF and separation was carried out with four columns packed with particle gels bearing different nominal pore sizes. The elution rate was 1 mL/min at 30 °C. The molecular weights were calculated based on a calibration curve from monodisperse polystyrene standards. 

Infrared spectroscopy (FT-IR) was performed with a Nicolet Nexus 670 (Wake Forest, NC, USA) spectrometer equipped with a single horizontal golden gate attenuated total reflectance (ATR) cell. Spectra were recorded by averaging 20 scans between 4000 and 400 cm^−1^ with a resolution of 2 cm^−1^ under ambient conditions.

^1^H-NMR and ^13^C-NMR spectroscopy were used in order to determine the composition along with the microstructure of the PI segments, as well as to verify the successful chemical modifications (hydrogenation and sulfonation). Samples were dissolved in deuterated chloroform (CDCl_3_). The spectra were recorded at room temperature on an Avance Bruker 500 MHz (Rheinstetten, Germany) equipped with a Bruker DSX NMR spectrometer with a BBO z-gradient probe using a rate of 5000 Hz, a frequency of 500 MHz and a delay of 1 s. 

A morphological characterization in order to evaluate the surface morphology of all thin film samples using different parameters (temperature and solvent) was accomplished with a Nanoscope IVa Dimension 3100 AFM (Digital Instruments, Plainview, NY, USA). A tapping mode in air was employed using an integrated silicon tip/cantilever (125 μm in length and with a resonant frequency of ca. 300 kHz) at a scan rate of 1.0 Hz and a resonance frequency of ~300 kHz. The measurements were performed with 512 scan lines. All samples were dissolved in different solvents (THF, toluene, cyclohexane), preparing 5 wt% solution concentrations using spin casting onto glass substrates in ambient conditions under specific conditions (2000 rpm for 60 s), which led to thicknesses ranging from 50 nm to 100 nm (depending on the evaporating solvent). After the complete evaporation of the solvents, the casted samples were annealed at the following temperatures: room temperature, 80 °C, 100 °C and 120 °C. 

For the cleaning procedure of the substrates, a piranha solution was used (H_2_O_2_ 30% and NH_4_OH 35%, 1:1:5 volume fractions of hydrogen peroxide solution, ammonium hydroxide solution, and water, respectively, for 1 h at 60 °C). In addition, the substrates were washed with pure ethanol and, prior to use, were placed in an UV/O_3_ chamber for approximately 30 min in order to remove any organic material residue.

The parameters used for the spin coating procedure were as follows: a spinning velocity of ~3000 rpm and a polymer solution concentration equal to 3% for approximately 30 s. 

The thin films were prepared by spin casting onto glass substrates under ambient conditions. In addition, the bulk samples were cast at ambient conditions for at least seven days in order to promote the thermodynamically stable phases.

The structure in the bulk films was studied with small-angle X-ray scattering (SAXS). The 2D X-ray patterns were measured with a Xenocs WAXS/SAXS X-ray (Grenoble, France) system using a Pilatus 300 K detector and a Genix3D X-ray source with a wavelength of 1.542 Å. The calibration, 1D reduction and analysis of the patterns were performed with home-made routines designed in Igor Pro software (WaveMetrics Inc. Portland, OR, USA). The values of the sample–detector distance and reciprocal space vector **s** (|s| = 2 sin (θ)/λ) were calibrated using several orders of the main scattering peak of a silver behenate powder. All bulk samples were prepared using a 5 wt % solution in THF and/or toluene prior to SAXS experiments.

## 3. Results and Discussion

### 3.1. Molecular Characterization

**Figure 1:**
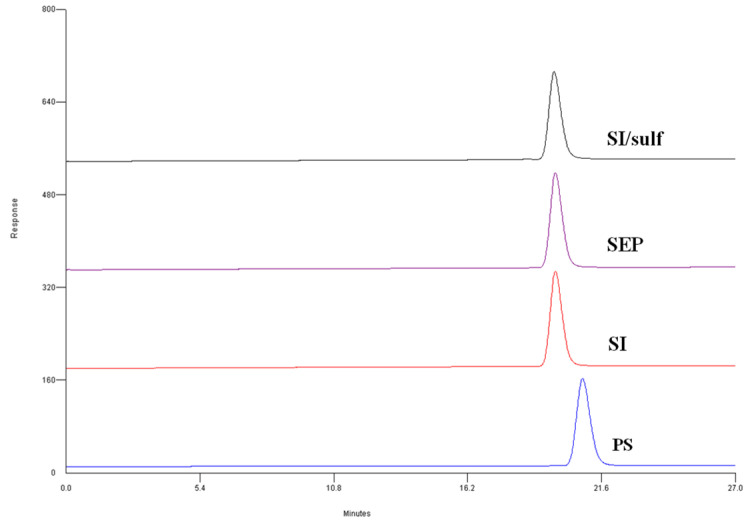
SEC chromatograms of the synthesized materials where the color blue corresponds to the PS homopolymer precursor, red to the SI diblock copolymer, purple to the hydrogenated SEP sample and black to the sulfonated SI/sulf copolymer.

*Size Exclusion Chromatography***.** The technique was employed in order to molecularly characterize the SI sample as well as the chemically modified materials, regarding their molecular homogeneity, narrow dispersity and the absence of any by-products that would be attributed to side reactions in the final modified BCPs. From SEC results, the total number-average molecular weights were calculated as equal to 56.7 kg/mol, exhibiting a narrow dispersity index (*Ð* = 1.04) for the initial BCP; specifically, the PS number average molecular weight was estimated to be 40.6 kg/mol, whereas it was estimated to be 16.1 kg/mol for the PI segment. In Figure 1, all chromatograms, including the PS homopolymer together with the SI, SEP and SI/sulf copolymers, are presented, verifying the absence of by products and the maintenance of the monomodal distributions at the same elution time even though the PI hydrodynamic radius is altered [23,24]. 

*Infrared Spectroscopy***.** This specific technique was used in order to identify the characteristic chemical groups evident in the SI, the SEP and the SI/sulf copolymers and to assess the successful outcome of the modification reactions. After sulfonation, the double bond (>C=C<) of the PI monomeric units were severely diminished, since the yield of the reaction was not 100%. In the case of complete hydrogenation, the carbon double bonds were eliminated. The different microstructures of the PI (−1,4: 839 cm^−1^/−3,4: 890 cm^−1^ and −1,2:910 cm^−1^) [17,49,50] showcased a similar behavior. Therefore, the band intensities in the modified materials were either decreased (after partial sulfonation) or completely disappeared (after full hydrogentation). Additionally, the attached sulfonyl groups to the PI modified monomeric units resulted in the appearance of new peaks attributed to the hydrophilic character, as indicated by the –OH groups of the amphiphilic SI/sulf copolymer.

**Figure 2:**
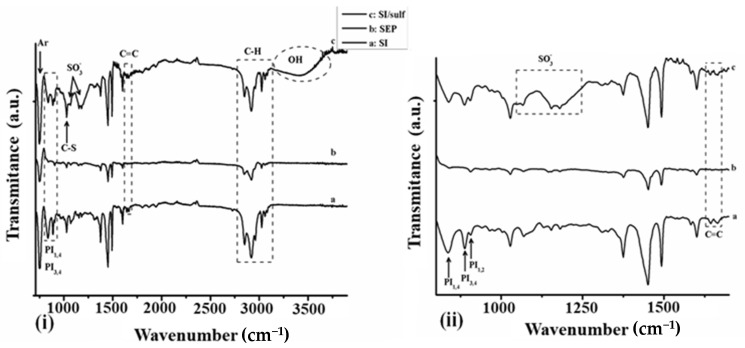
FT-IR spectra of (a) SI, (b) SEP and (c) SI/sulf copolymers indicating the characteristic groups as well as the differences after chemical modification reactions. The spectra in (**i**) correspond to wavelengths from 750 to 3750 cm^−1^ and in (**ii**) to magnified areas from 750 to 1750 cm^−1^.

In Figure 2i the FT-IR spectra for all samples are presented and the expected alternations are indicated. In Figure 2ii, a magnified area of the FT-IR spectra is illustrated in order to highlight the differences in the characteristic groups for the initial copolymer and the respective derivatives. Furthermore, in Table S1 (in the Supporting Information), the characteristic peaks of all samples are presented in comparison with the theoretically predicted peaks.

Analyzing the data from the FT-IR spectra, important results regarding the chemical modifications can be extracted. The >C=C< bond as well as the >C-H bond on the aromatic ring can be clearly identified at 1500 cm^−1^, 1600 cm^−1^ and 760 cm^−1^, respectively [18], whereas after 3000 cm^−1^, the C-H vibrations are evident [18]. The characteristic stretching of the double bond of the PI block at 1664 cm^−1^ [18] as well as the characteristic peaks for the different microstructures (−1,4, −1,2 and −3,4) at the regime of 839–910 cm^−1^ [49] are evident. Furthermore, the presence of fewer peaks in the area of 800–1000 cm^−1^ after hydrogenation, are observed [19], whereas for the SI/sulf sample, new intense peaks emerged due to the sulfonation. As far as the sulfonated sample is concerned, two different types of peaks attributed to the sulfonyl group (SO_3_^–^) are evident (symmetric vibrations at 1042 cm^−1^ and antisymmetric vibrations at 1184 cm^−1^). Finally, the presence of -OH stretching vibrations at 3500 cm^−1^ (due to humidity) further confirm the amphiphilic character of the sulfonated material as expected [51]. 

*Proton Nuclear Magnetic Resonance (^1^H-NMR) and Carbon Nuclear Magnetic Resonance (^13^C-NMR) Spectroscopy*. Nuclear magnetic resonance spectroscopy was applied in order to determine the composition of each segment as well as the volume fractions, the PI microstructures, and the degree of hydrogenation and sulfonation. The volume fractions were calculated from ^1^H-NMR mass fractions, using the following block densities: d_PS_ = 1.06 g/mL and d_PI_ = 0.93 g/mL. 

**Figure 3:**
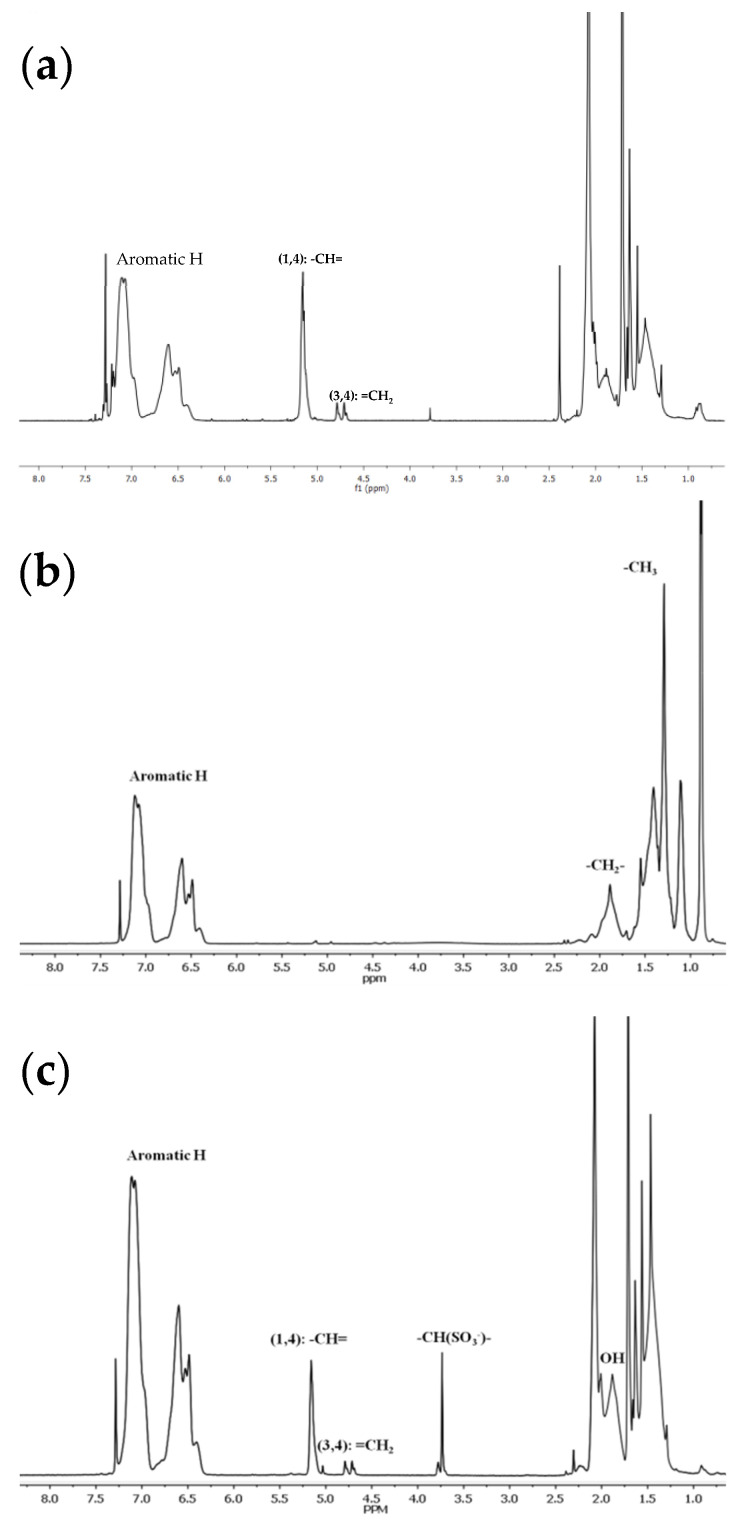
^1^H-NMR spectra of the synthesized materials corresponding to: (**a**) the initial pristine SI diblock copolymer, (**b**) the hydrogenated SEP sample indicating an almost 100% hydrogenation degree and (**c**) the partially sulfonated SI/sulf copolymer indicating an approximately 43% sulfonation degree of the PI domains.

The ^1^H-NMR spectra of the initial copolymers, as well as the hydrogenated and the sulfonated copolymers, are presented in Figure 3. In Figure 3a, the characteristic chemical shifts at 5.10 parts per million (ppm) are attributed to the one proton of the monomeric unit of the PI_1,4_ microstructure, whereas those at 4.70 ppm are assigned to the two protons of the monomeric units of the PI_3,4_ microstructure for the initial diblock copolymer. In Table S2 (in the Supporting Information), the theoretically expected chemical shifts along with the experimentally observed shifts are given.

Concerning the ^1^H-NMR spectrum of the hydrogenated material (Figure 3b), the major difference lays upon the complete saturation of the carbon double bonds, which is confirmed by the total elimination of the peaks corresponding to the -H of the double bond for both −1,4 and −3,4, microstructures, indicating an almost 100% degree of hydrogenation [14,17,18,52,53]. As is evident in Figure 3b, the chemical shifts corresponding to the PI block are clearly eliminated when compared to the initial spectrum of the copolymer ^1^H-NMR (Figure 3a). The intense chemical shifts at the region between 0.50 and 2.00 ppm are attributed to PP (polypropylene) and PE (polyethylene) (methylene and/or methyl units) after the complete hydrogenation of the PI [17,50,52].

The ^1^H-NMR spectrum of the sulfonated block copolymer (Figure 3c) indicated new chemical shifts due to the alteration of the chemical structure and the presence of the sulfonyl groups (-C**H**(SO_3_^−^)-) in some of the PI monomeric units. These shifts are located at 3.75 ppm, indicating the partially sulfonated PI chains, whereas the presence of hydroxyl groups at 2.00 ppm was also obvious. The incomplete sulfonation reaction was verified by the existence of the characteristic chemical shifts of the PI segment at ~5.00 ppm. Taking into consideration the total number-average molecular weight, as well as the mass fractions for both the SI and SI/sulf (5.10 ppm for the PI and 3.75 ppm for the sulfonated PI), a sulfonation degree of approximately 43% was estimated.

The verification of the characteristic carbon groups attributed to the sulfonyl groups of the modified material was achieved through ^13^C-NMR (Figure S1 in the Supporting Information), since the characteristic chemical shifts of the –C(CH_3_)(OH)- group at 76.0 ppm and –CH(SO_3_^−^)- at 78.0 ppm strongly indicated the partially successful sulfonation (up to 43%). It is worth mentioning that the characteristic shifts corresponding to the 1,4-PI microstructure at 125.0/135.0 ppm and to the 3,4-PI microstructure at 126.0/143.0 ppm are not evident, as can be observed in Figure S1. Furthermore, in Table S3, the theoretically predicted and experimentally observed chemical shifts attributed to the different chemical groups are presented. 

### 3.2. Morphological Characterization

Bulk self-assembly morphologies for the diblock copolymer of the SI type have been thoroughly studied and analyzed in the literature [54]. Even though the use of hydrogenated and/or sulfonated materials for membrane applications has been reported in the literature [31,36,37,38,39], the surface behavior regarding thin film preparation and the self-assembly of the modified materials has drawn less attention. Different treatment parameters were employed in order to determine the structure–properties relationship of all samples using various organic solvents (toluene, THF and cyclohexane) at multiple annealing temperatures (room temperature, 80 °C, 100 °C and 120 °C) utilizing a spin casting method eventually leading to various surface morphologies. Furthermore, the structure evolution under thermal annealing for all copolymers was studied in bulk using SAXS. Both AFM and SAXS results showcased well-ordered structures, indicating the absence of any homopolymer and/or by-product traces. 

Different adjustable parameters such as total average molecular weight, structure, composition, conformation, environmental parameters and processing conditions hold a key role in the dissolution of polymeric materials [55] and determine the final adopted morphologies. According to Hansen theory [56], the overall solubility is governed by the following equation:

δ^2^ = δ_d_^2^ + δ_p_^2^ + δ_h_^2^ (1)

where (δ_d_) corresponds to dispersion solubility, (δ_p_) to the dipole solubility and (δ_h_) to hydrogen bond solubility. Furthermore, these parameters are related to the Flory–Huggins parameter *χ*, which indicates whether the system will be microphase separated, as can be realized from the equation

*χ*_AB_ ≈ (δ_A −_ δ_B_)^2^V_reff_/RT (2)

and among solvents and polymers, as proposed by Equation (3). On the other hand, Equation (4) is suggested by Hansen:

*χ*_sp_ = 0.34 + (δ_s_ − δ_p_)V_s_/RT (3)

*χ*_sp_ = (aV/RT)[(δ_ds_ − δ_dp_)^2^ + 0.25(δ_ps −_ δ_pp_)^2^ + 0.25(δ_hs_ − δ_hp_)^2^] (4)

The lower the *χ*_sp_ parameter, the better the affinity and, subsequently, the better the mixing, whereas temperature increases result in even smaller values of *χ*_sp_, eventually leading to mixed components [55,56]. 

For less studied polymers, the parameters δ_d_, δ_p_ and δ_h_ can be calculated from equations utilizing Hoftyzer and Van Krevelen methods [57], which take into consideration the group contributions F_di_, F_pi_ and E_hi_:

δ_d_ = ΣF_di_/V (5)

δ_p_ = √ΣF_pi_^2^/V (6)

δ_h_ = √ΣE_di_/√V (7)

Additionally, the polymer cohesive energy (E_coh_) can be directly calculated by a similar method where the solubility parameter can be found using the equation

Δ = (E_coh_/V)^1/2^ (8)

Both methods (Hansen and Van Krevelen) were used in order to determine the δ parameter of the sulfonated PI block. In Table S4 (in the Supporting Information), the results from the aforementioned equations for the δ parameter are given.

From Table S4 it can be concluded that the calculated δ parameter that exploits the two different methods for both solvents and polymer blocks is coherent. The deviations found in the SEP and SI/sulf samples are attributed to the calculation method as well as to the strong polar and hydrogen bonding forces, especially for the sulfonated and non-sulfonated PI monomeric units. Taking into consideration correlated studies in the literature [56], similar sulfonated materials exhibit relatively high δ_p_ and δ_h_ such as chlorosulfonated polyethylene (4.7 and 4.9 MPa), polysulfone (8.3 and 8.3 MPa) and polysulphide rubber (17.3 and 16.7 MPa).

In order to draw possible conclusions regarding the affinity among the blocks involved in this study, Equation (2) was utilized and through the comparison of δ parameters of all synthesized segments, it was obvious that the PI/sulf presented less affinity towards the other blocks. In Table 1, the results regarding the affinity among the abovementioned blocks in different temperatures are presented.

All *χ*_AB_ values were calculated from Equation (2), where V_reff_ = 100 cm^3^/mol, R = 8.314 cm^3^ MPa/K·mol and δ in MPa, as calculated using the Hansen equation. It should be noted that the values are significantly different (lower) at least for the pristine diblock copolymer based on experimental data, as reported in the literature [54,58,59,60]. The theoretical calculations are being reported for all different systems for comparison.

It should be noted that due to the partial sulfonation in the case of SI/sulf, three different chemical structures were observed, namely PS, PI and PI/sulf. As a result, the interaction parameter between PI and PI/sulf should not be overlooked. Moreover, the conclusions derived from this study are supported by the literature regarding the *χ* parameter which exhibits higher value in the case of PS/PEP [8,9,61]. 

The affinity of each solvent used, namely THF, toluene and cyclohexane, strongly affects the *χ*_SP_ of each block and taking advantage of Equation (4) along with the δ_d_ (solvent/polymer dispersion solubility), δ_p_ (solvent/polymer polar solubility) and δ_h_ (solvent/polymer hydrogen bonding solubility), *χ*_SP_ values were estimated and are presented already in the literature by Politakos et al. [47]. 

More specifically, the PS segments present higher affinity towards THF and slightly less towards toluene and cyclohexane, whereas PI and PEP blocks exhibit higher affinity towards toluene and satisfactory enough towards cyclohexane and THF. PI/sulf blocks display higher affinity in the presence of THF which is attributed to the dispersion forces, the polar and hydrogen bonding as well as to the chemical structure of both polymer and solvent. THF polar character in conjunction with the hydrogen bonding capability render the solvent ideal for the PI/sulf blocks when compared to non-polar solvents such as toluene and cyclohexane, which are preferable for the non-polar polymer blocks (e.g., PI and PEP). As a general remark regarding the THF solvent, apart from the affinity (due to the polarity), another critical parameter is the quick evaporation rate when compared to toluene and cyclohexane, which offers less time for the blocks to self-assemble. 

In order to justify the homogeneous surface type topology in the prepared thin films we have calculated from the AFM images the respective roughness (nm), skewness and kurtosis, and the data are illustrated in Table S5 in the Supporting Information. The analysis of the calculated parameters leads to the fact that, according to the solvent used during the spin casting procedure (cyclohexane, THF and toluene) as well as the annealing temperature (R.T., 80 °C, 100 °C, and 120 °C), the values show a specific trend according to the adopted structure in each case. Further confirmation of the adopted morphologies using toluene as the casting solvent (R.T., 80 °C) was achieved by the power spectral density (PSD) calculation results for specific samples, as evident in Figure S3 (Supporting Information).

### 3.3. Initial Diblock Copolymer (PS-b-PI or SI) 

**Figure 4:**
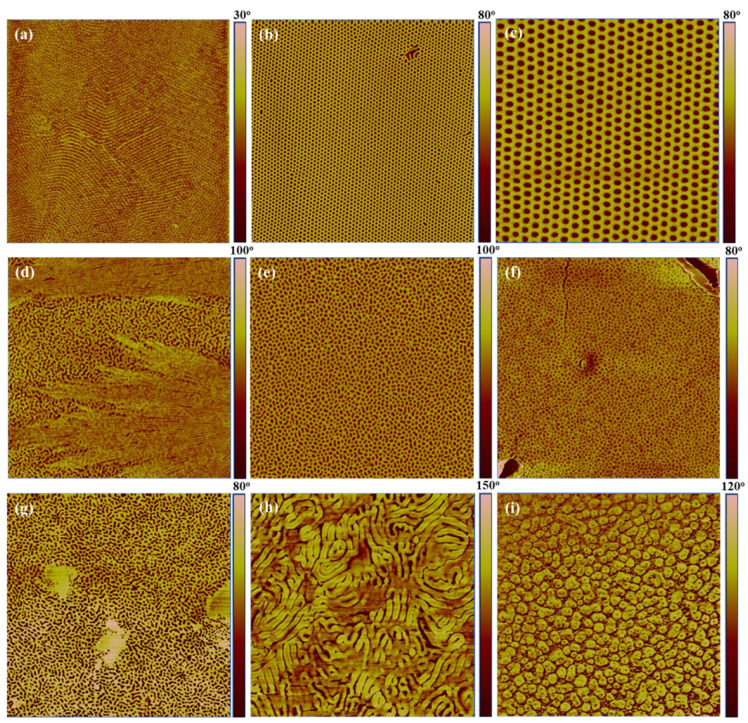
AFM phase images (in all images, the scale is 3 μm × 3 μm except for Figure 4c, where the scale is 1 μm × 1 μm) corresponding to: (**a**) the pristine initial diblock copolymer (SI) where hexagonally close-packed cylinders parallel to the substrate surface are observed (toluene/R.T.); (**b**–**g**) the 100% hydrogenated (SEP) material where (**b**,**c**) well-ordered hexagonally close-packed cylinders are evident (toluene/R.T.); (**d**) sponge-like structures in combination with crystallites are obvious (cyclohexane/80 °C); (**e**) hexagonally close-packed cylindrical topology is obtained (THF/80 °C); (**f**,**g**) disordered structures are observed (THF/100 °C and 120 °C, respectively); (**h**,**i**) the partially sulfonated (SI/sulf) copolymer where (**h**) horizontal cylinders (cyclohexane/R.T.) and (**i**) a micellar structure (cyclohexane/80 °C) are evident.

**Figure 5:**
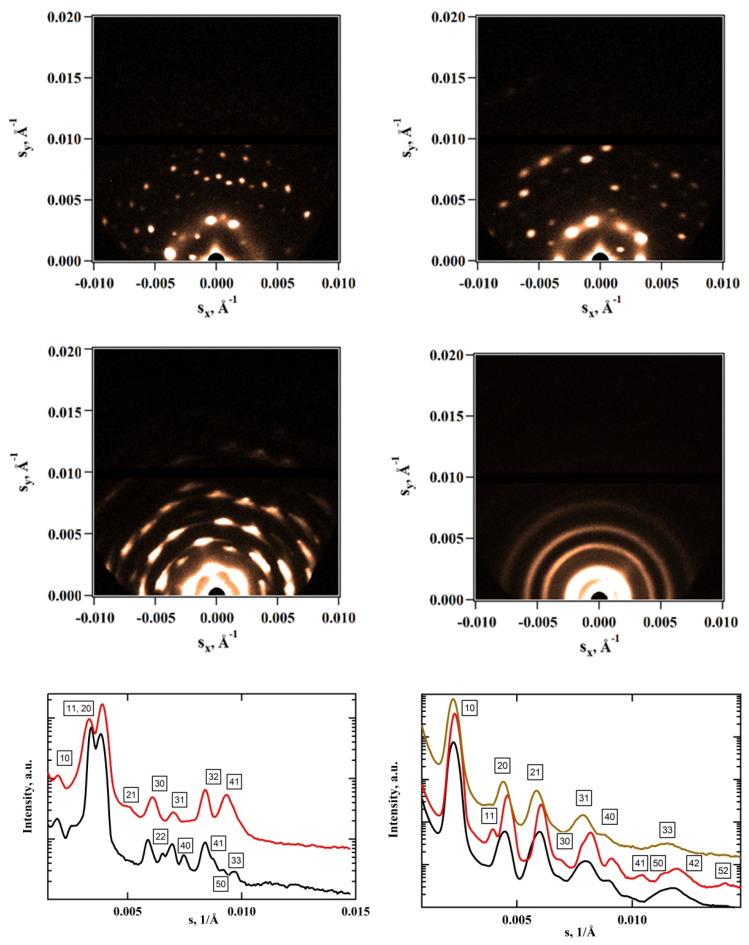
Two-dimensional SAXS patterns of the sample SI cast from THF at R.T. (**top left**) and subsequently annealed at 80 °C (**top right**), as well as the sample SEP cast from THF at R.T. and subsequently annealed at 80 °C (**middle left**), and the same sample annealed at 120 °C (**middle right**). The 1D-reduced data corresponding to samples SI and SEP described above are given in the **bottom left** and **bottom right** panels, respectively. In the bottom graphs, the as-prepared samples are shown in black, the ones annealed at 80 °C in red and at 120 °C in brown. The *hk* indices of the hexagonal lattice are indicated in 1D curves.

Due to the low *χ* parameter for the SI system, the use of different solvents at various temperatures led in most cases to significantly less formed structures. Using toluene as a casting solvent at room temperature employing the spin casting method led to predominantly hexagonally close-packed cylinders parallel to the substrate surface (Figure 4a). These are not very well ordered, probably due to the high evaporation speed during the casting process. Additional AFM images for the pristine SI sample for different casting solvents and annealing temperatures are presented in Figure S2a,b (in the Supporting Information), specifically for toluene and cyclohexane at 80 °C for comparison. The 2D PSD function (Figure S3, left) calculated for the image in Figure 4a shows the presence of only one diffraction peak located at 42.5 nm. After annealing the sample at 80 °C, the structure did not show any significant improvement (cf. Figure S2a), which is reflected in the corresponding 2D PSD function (Figure S3, middle). Indeed, the diffraction peak remains broad and does not show any orientation, whereas its characteristic distance slightly reduces to 40.6 nm. The further characterization of the sample structure has been performed with SAXS. The 2D patterns of the sample cast from THF and the one annealed at 80 °C (Figure 5 at the top left and top right, respectively) exhibit point-like reflexes, which is indicative of large-size monodomains. The indexation of the patterns to a hexagonal unit cell (cf. Figure 5, bottom left) shows a nice agreement between the experimental and calculated peak positions (Figure S4, left), providing the lattice parameter of 61.1 and 58.6 nm, respectively. Therefore, according to SAXS the lattice shrinks upon annealing by about 4%. This trend is also shown by AFM; however, the uncertainty in the crystallography of the observed plane makes the calculation of the lattice parameter relatively imprecise. Thus, if one assumes that the AFM image captures ten crystallographic planes, the relative error in the determination of the lattice parameter will be about 24%. 

### 3.4. Hydrogenated Diblock Copolymer (SEP)

The 100% hydrogenation of the PI block from the initial pristine diblock resulted in an increase in the *χ* parameter value (as calculated theoretically by the Hansen equation, Table 1) if we take into consideration the better ordered structures. Well-ordered hexagonally close-packed cylinders were obtained for the SEP sample when diluted in toluene at room temperature, as evident in Figure 4b,c. The PSD function calculated from the AFM image in Figure 4b shows a nice hexagonal pattern in the reciprocal space (Figure S3, right), which confirms the highly organized structure visible in the image. The corresponding lattice parameter calculated from the Fourier transform equals 44.2 nm. The SAXS data corresponding to the as-prepared film confirms the hexagonal structure of SEP (cf. Figure 5, bottom right) with the corresponding lattice parameter equal to 51.5 nm. Therefore, the AFM image provides an excellent estimate of the lattice parameter (51.0 nm), which shows that when the structure is characterized by long-range order and the crystallography of the surface is known, the image can provide a very good estimation of the lattice parameter. 

When cyclohexane was used at room temperature, the obtained morphology was different and can be ascribed as a disordered micellar structure probably due to the higher interaction parameter values between the PS and poly(ethylene-*alt*-propylene) domains. In the case of an annealing temperature equal to 80 °C and the use of cyclohexane as the solvent, the adopted topology was further modified, exhibiting sponge-like structures in combination with crystallites (Figure 4d) due to the existence of the PEP segments (the initiation of crystallization is possible at 80 °C or below, based on the molecular characteristics). 

Similarly, by altering the solvent and annealing temperature (THF, 80 °C), a hexagonally close-packed cylindrical topology was obtained as depicted in Figure 4e, which is becoming a disordered structure as the annealing temperature increases to 100 °C (Figure 4f) and 120 °C (Figure 4g) as expected, since the chi parameter values decreased, as evident in Table 1. The SAXS data nicely corroborate this picture. Indeed, the 2D SAXS pattern of the SEP sample (THF, 80 °C) shows hexagonal packing with a relatively large size of monodomains (Figure 5, middle left), which is evident from the point-like appearance of the peaks. By contrast, upon annealing at 120 °C, the pattern significantly modifies (Figure 5, middle right). Indeed, now the point-like reflexes are absent and replaced by almost uniform rings, which testifies that the monodomain size is drastically reduced. Interestingly, the change in the sample structure can also be monitored from the lattice parameter *a*. The annealing of the sample at 80 °C brings about a relatively small decrease in *a* from 51.1 to 50.9 nm, whereas the annealing at 120 °C causes a more noticeable increase in *a* to 52.5 nm, which is in line with the sample disordering.

### 3.5. Partially Sulfonated Diblock Copolymer (SI/Sulf)

The partial sulfonation of the initial PI chains with a yield of 43% resulted in the calculation of not just one Flory–Huggins interaction parameter but two: *χ*_PS/PIsulf_ and *χ*_PI/PIsulf_. The latter parameter is the one between non-sulfonated and sulfonated monomeric units in the same PI macromolecules, possibly rendering the specific modified final copolymer less susceptible to dissolution in non-polar solvents. The partially sulfonated PI blocks induced the possible formation of horizontal cylinders (Figure 4h) when cast from cyclohexane at room temperature and were further altered to a possible micellar structure when the sample was annealed at 80 °C (Figure 4i) under the same solvent casting conditions. A similar morphology was observed when using toluene as the casting solvent at temperatures ranging from 80 °C to 120 °C, as evident in Figure S2c,d (in the Supporting Information). 

## 4. Conclusions

We report the synthesis of the polystyrene-*b*-poly(isoprene) diblock copolymer through anionic polymerization and the subsequent chemical modifications towards the formation of either the polystyrene-*b*-poly[(ethylene)-*alt*-(propylene)] copolymer or polystyrene-*b*-poly(sulfonated isoprene-*co*-isoprene). The chemical modification of SI block copolymer was achieved i) through almost 100% hydrogenation of the PI block in order to obtain a SEP copolymer and ii) through the sulfonation reaction of the PI obtaining a partially sulfonated PI block (43% yield). 

All copolymers were molecularly characterized through various techniques in order to verify the successful synthesis and post-polymerization modifications. The chemically modified copolymers were characterized via SEC in order to justify the absence of any undesired side reactions. For the verification of the synthesized SEP and SI/sulf samples, FT-IR, ^1^H-NMR and ^13^C-NMR techniques were used. A fully hydrogenated material was obtained since the characteristic chemical shifts attributed to the PI_1,4_ and PI_3,4_ were completely eliminated while the degree of sulfonation was calculated at approximately 43% through ^1^H-NMR. This led to a block copolymer containing PS and coexistent PI sulfonated and non-sulfonated monomeric units. 

The structural characterization on the thin film self-assembly of both the copolymer precursor and the final modified materials is reported for the first time to the best of our knowledge. By adopting the spin casting method under specific conditions and varying significant parameters such as solvent (toluene, THF, cyclohexane) and annealing temperature (RT, 80 °C, 100 °C and 120 °C) different topologies were justified when studied with atomic force microscopy. It is evident that the pristine copolymer had less well-ordered structures probably due to the fast evaporation rate of the solvents used during the spin casting and the fact that the samples were only annealed for short periods. The highest repulsion among PS and PEP chains led to the formation of long-range morphologies using different solvents and annealing temperatures. For the SI/sulf system, morphologies ranging from disordered to spherical micelles, and even to cylindrical structures, were observed deriving from the process treatment along with the amphiphilic character of the specific material. 

The results obtained from AFM were further supported by small-angle X-ray scattering experiments, as could be clearly observed on the 2D power spectral density data. The different adopted nanostructures in bulk were determined using SAXS and the effect of thermal annealing on the obtained morphologies as well as on *d*-spacing were justified. 

## Figures and Tables

**Table 1:** Characteristic results for the *χ*_AB_ between all block combinations concerning different chemical structures at different temperatures.

*χ*_AB_ → T(K)	300 K	353 K	373 K	393 K
*χ*_PS/PI_	0.051	0.043	0.040	0.039
*χ*_PS/PEP_	0.102	0.087	0.082	0.079
*χ*_PS/PIsulf_	1.350	1.180	1.080	1.046
*χ*_PI/PIsulf_	1.927	1.638	1.541	1.493

## Data Availability

The data presented in this study are available upon request from the corresponding authors.

## Supplementary Materials

The following are available online at www.mdpi.com/article/10.3390/polym13234167/s1, Table S1: Characteristic FT-IR peak wavenumbers for SI, SEP and SI/sulf block copolymers, Table S2: Characteristic chemical groups together with the corresponding theoretical and experimental chemical shifts for the SI, SEP and SI/sulf block copolymers, Figure S1: ^13^C-NMR spectrum for the sulfonated sample SI/sulf where the characteristic chemical shifts justify the successful partial sulfonation procedure, Table S3: Characteristic chemical groups together with the corresponding theoretical and experimental chemical shifts for the SI/sulf block copolymer, Table S4: Characteristic solubility parameters and volume values for each solvent and polymer segment that have been used in order to extract Hansen and Van Krevelen solubility parameters, Table S5: The roughness (nm), skewness and kurtosis as calculated from the AFM images for the initial SI sample, the hydrogenated (PEP) and the sulfonated (SI/sulf) copolymers using cyclohexane, tetrahydrofuran and toluene as casting solvents at different annealing temperatures (RT, 80 °C, 100 °C and 120 °C), Figure S2: AFM phase images (3 μm × 3 μm except) corresponding to: the pristine initial diblock copolymer (SI) in (a) toluene/80 °C, (b) cyclohexane/80 °C and to the partially sulfonated (SI/sulf) copolymer (c,d) where possible micellar structures are illustrated in toluene/100 °C (c) and toluene/120 °C (d), respectively, Figure S3: 2D Power spectral density functions corresponding to AFM images (Figure 4a,b and Figure S2a) of the pristine initial diblock copolymer (SI) cast from toluene at R.T. (left), the same sample after annealing at 80 °C (middle) and the 100% hydrogenated (SEP) material in the as-prepared state (right). The dashed green circles are tranced through the fundamental diffraction peak, Figure S4: Comparison of the experimental and calculated d-spacings for samples SI cast from toluene at R.T. and upon annealing at 80 °C (left) and the SEP material cast from THF at R.T. and upon annealing at 80 °C and 120 °C (right). The solid lines correspond to equation y = x. (See main text for more details).
