# Supplementary material for: Molecular and Structure–Properties Comparison of an Anionically Synthesized Diblock Copolymer of the PS-b-PI Sequence and Its Hydrogenated or Sulfonated Derivatives
Source: Polymers (Basel). 2021 Nov 28;13(23):4167. doi: 10.3390/polym13234167 (PMC8659476; doi:10.3390/polym13234167)
Supplement: Supplementary file 1 [file polymers-13-04167-s001.zip › polymers-1479408-supplementary.pdf]

Molecular and Structure/Properties Comparison of an Anionically Synthesized Diblock Copolymer of the PS-*b*-PI Sequence and its Hydrogenated or Sulfonated Derivatives

Nikolaos Politakos[a,b], Gkreti-Maria Manesi[a], Ioannis Moutsios[a], Konstantinos Artopoiadis[a], Konstantina Tsitoni[a], Dimitrios Moschovas[a], Alexey A. Piryazev[c,d], Denis S. Kotlyarskiy[c,d], Galder Kortaberria[e], Dimitri A. Ivanov[c,d,f], Apostolos Avgeropoulos[a,c,*]

[a] Department of Materials Science Engineering, University of Ioannina, University Campus-Dourouti, 45110 Ioannina, Greece

[b] POLYMAT and Departamento de Química Aplicada, Facultad de Ciencias Químicas, University of the Basque Country UPV/EHU, Joxe Mari Korta zentroa, Tolosa etorbidea 72, Donostia-San Sebastián, 20018, Spain

[c] Faculty of Chemistry, Lomonosov Moscow State University (MSU), GSP-1, 1-3 Leninskiye Gory, 119991 Moscow, Russia

[d] Institute of Problems of Chemical Physics, Russian Academy of Sciences, Chernogolovka, 142432 Moscow, Russia

[e] 'Materials+Tecnologies' Research Group, Chemistry and Environmental Engineering Department, Faculty of Engineering, Gipuzkoa, University of the Basque Country (UPV/EHU), Plaza Europa 1, 20018 Donostia, Spain

[f] Institut de Sciences des Matériaux de Mulhouse – IS2M, CNRS UMR7361, 15 Jean Starcky, Mulhouse 68057, France

*Corresponding author: Professor Dimitri A. Ivanov, Professor Apostolos Avgeropoulos
E-mail address: dimitri.ivanov@uha.fr, aavger@uoi.gr

Supporting Information

The following data are given in the Supporting Information:

(A) Synthetic and chemical modification reactions protocol.

(B) Characteristic wavenumbers for specific chemical groups of all samples (initial copolymer and the chemical modified ones) from FT-IR spectroscopy (Table S1)

(C) ^{1}H-NMR & ^{13}C-NMR Spectroscopy Molecular Characterization Results (Table S2, Table S3 and Figure S1)

(D) Estimation of solubility parameters (Table S4)

(E) AFM characterization results (Table S5, Figure S2)

(F) SAXS Results (Figure S3, Figure S4)

(A) Synthetic and chemical modification reactions protocol.

For the synthesis of the PS block, 4g of styrene (0.038 mol) were initiated using *sec*-BuLi (0,09 mmol) in a non-polar solvent (benzene, 200 ml) and after the complete polymerization of the first block, 1,5g of isoprene (0.022 mol) was added to the solution and the reaction was completed after 24 hours. For the hydrogenation, 2.4g of the initial PS-*b*-PI sample were dissolved in 40 ml of p-xylene and were introduced to 24g of p-toluenesulfonyl hydrazide (109 mmol) at 135°C for 3 hours. The hydrogenated sample was then precipitated twice in cool methanol and was dried under vacuum. For the sulfonation 2g of anydrous sulfuric acid was added dropwise under stirring to 20 g of 1,4-dioxane. A different solution containing 2 g of the initial diblock copolymer and 20 g of 1,4-dioxane was prepared and the sulfur trioxide/1,4 dioxane complex solution was added at 25°C for 2 hours. The neutralization of the solution was accomplished using 15 wt % of NaOH followed by the addition of methanol and the final solution was heat at 80°C for 4 hours under stirring. The organic solvents were removed under high vacuum.

(B) Characteristic wavenumbers for specific chemical groups of all samples (initial copolymer and the chemical modified ones) from FT-IR spectroscopy.

Table S1: Characteristic FT-IR peak wavenumbers for SI, SEP and SI/sulf block copolymers.

Chemical groups	Wavenumber (cm^{-1})	SI	SEP	SI/sulf
**C-H aromatic**	760	√	√	√
**C-H**	3000>	√	√	√
**>C=C< stretching**	1664	√	-	~
**$PI_{1,4}$/$PI_{3,4}$/$PI_{1,2}$**	839/890/910	√	-	~
**C=C aromatic**	1500,1600	√	√	√
**C-S**	1000-1250	-	-	√
**SO_3^- symmetric**	1042	-	-	√
**SO_3^- antisymmetric**	1184	-	-	√
**OH stretching of H_2O**	3500	-	-	√

A. √: Existence of the band
B. ~: Existence with lower intensity
C. -: Not evident

(C) ^{1}H-NMR & ^{13}C-NMR Spectroscopy Molecular Characterization Results

Table S2: Characteristic chemical groups together with the corresponding theoretical and experimental chemical shifts for the SI, SEP and SI/sulf block copolymers.

Chemical groups	Chemical shifts (ppm)	SI	SEP	SI/sulf
**$-CH_3$**	1.64	√	√	√
**$-CH_2-$**	2.01	√	√	√
**-CH=C- ($PI_{1,4}$)**	5.10	√	-	√*
**$=CH_2$ ($PI_{3,4}$)**	4.70	√	-	√*
**Aromatic region**	6.75-7.25	√	√	√
**OH**	2.00	-	-	√
**$-CH(SO_3^-)-$**	3.75	-	-	√

D. √: Existence of chemical shift

E. -: Not evident
F. *: Existence of the chemical shifts of the $PI_{1,4}$ and $PI_{3,4}$ indicated not complete sulfonation of the PI block

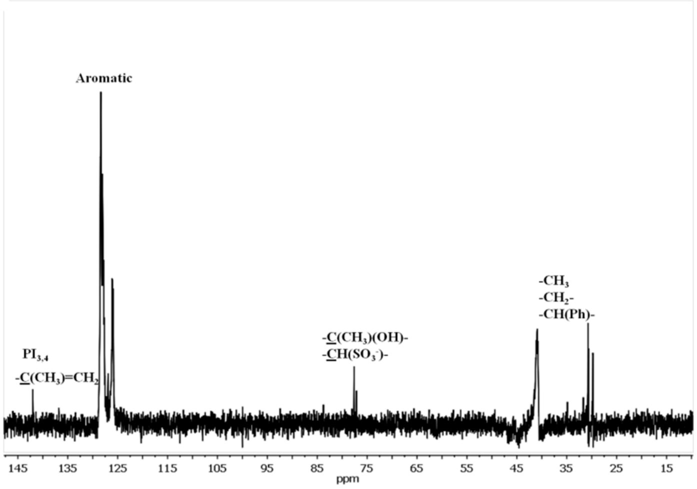

Figure S1: ^{13}C-NMR spectrum for the sulfonated sample SI/sulf where the characteristic chemical shifts justify the successful partial sulfonation procedure.

Table S3: Characteristic chemical groups together with the corresponding theoretical and experimental chemical shifts for the SI/sulf block copolymer.

Chemical groups	Chemical shifts (ppm) SI	Chemical shifts (ppm) SI/sulf	SI/sulf
**-CH_3**	23.0	24.0	√
**-CH_2-**	25.0-35.0	17.0-40.0	√
**-CH(Ph)-**	47.0	40.0	√
**-$CH=C(CH_3)$- ($PI_{1,4}$)**	125.0/135.0	-/-	-
**-$C(CH_3)=CH_2$ ($PI_{3,4}$)**	143.0/111.0	143/-	√**
**Aromatic C**	126.0-140.0	126.0-140.0	√
**-$C(CH_3)(OH)$-**	-	~76.0	√
**-$CH(SO_3^-)$-**	-	~78.0	√

√: Existence of the band
-: Not evident
**: Existence the chemical shift of the one C from the $PI_{3,4}$

(D) Estimation of solubility parameters

Table S4: Characteristic solubility parameters and volume values for each solvent and polymer segment that have been used in order to extract Hansen and Van Krevelen solubility parameters.

	δ_d (MPa)	δ_p (MPa)	δ_h (MPa)	V (cm^3/mol)	δ (MPa)[c] (Hansen)	δ (MPa)[d] (Van Krelen)
			**Solvents**			
**THF**	16.8	5.7	8.0	81.7	19.46	19.50
**Toluene**	18.0	1.4	2.0	106.8	18.16	18.20
**Cyclohexane**	16.8	0.0	0.2	108.7	16.80	16.70
			**Polymer blocks**			
**PS**	17.6	6.1	4.1	98.0	19.07	19.10
**PI**	17.4	3.1	3.1	75.7	17.94	17.40
**PEP**	16.7	0	5.2	80.8[b]	17.47	16.86[e]
**PI/sulf**	18.8[a]	5.8[a]	15.2[a,f]	86.1[b]	24.88	26.44[e]

[a] The solubility values were calculated for the sulfonated PI from the equations: (4a),(4b) and (4c) [1]. The remaining solubility values were determined based on the literature [2].

[b] The V values were calculated from the contribution on the total volume for each group of the chemical structure [1].

[c] The overall solubility was calculated from equation (1) [2].

[d] The solubility values were calculated from Van Krelen equation as reported in literature [1].

[e] The solubility was calculated from equation (5), by using the E_{coh} contributions for each group [1].

[f] The hydrogen bonding solubility (δ_h) was calculated without the contribution of the S atom, since no value was found in the literature [1].

(E) AFM characterization Results

Table S5: The roughness (nm), skewness and kurtosis as calculated from the AFM images for the initial SI sample, the hydrogenated (PEP) and the sulfonated (SI/sulf) copolymers using cyclohexane, tetrahydrofuran and toluene as casting solvents at different annealing temperatures (RT, 80°C, 100°C and 120°C).

Sample	Roughness (R_a:nm)	Skewness	Kurtosis
SI-RT-Cyclohexane	2.98	0.07	-0.16
SI-80-Cyclohexane	3.32	0.70	1.80
SI-100-Cyclohexane	4.46	0.61	0.32
SI-120-Cyclohexane	13.6	1.25	1.84
SI-RT-Tetrahydrofuran	5.20	0.40	1.02
SI-80-Tetrahydrofuran	8.10	0.02	-0.99
SI-100-Tetrahydrofuran	11.60	-0.80	0.07
SI-120-Tetrahydrofuran	21.80	-1.09	1.04
SI-RT-Toluene	1.40	0.29	0.34
SI-80-Toluene	1.95	7.75	4.70
SI-100-Toluene	2.73	1.64	5.21
SI-120-Toluene	12.70	0.28	0.08
PEP-RT-Cyclohexane	3.19	0.10	0.06
PEP-80-Cyclohexane	3.36	1.49	7.09
PEP-100-Cyclohexane	4.07	0.27	0.05
PEP-120-Cyclohexane	5.05	0.17	0.08
PEP-RT-Tetrahydrofuran	9.40	-0.05	0.29
PEP-80-Tetrahydrofuran	9.30	-0.76	0.51
PEP-100-Tetrahydrofuran	9.50	-0.30	0.13
PEP-120-Tetrahydrofuran	9.39	0.60	0.51
PEP-RT-Toluene	2.84	1.70	10.60
PEP-80-Toluene	7.06	0.90	0.90
PEP-100-Toluene	7.63	0.30	0.63
PEP-120-Toluene	11.05	-0.46	-0.06
SI/sulf-RT-Cyclohexane	8.60	2.2	7.51
SI/sulf-80-Cyclohexane	9.10	-1.52	1.66
SI/sulf-100-Cyclohexane	14.90	1.83	6.16
SI/sulf-120-Cyclohexane	17.75	2.55	6.71
SI/sulf-RT-Tetrahydrofuran	7.30	-1.11	1.84
SI/sulf-80-Tetrahydrofuran	4.78	-0.35	1.03
SI/sulf-100-Tetrahydrofuran	5.58	-2.61	0.88
SI/sulf-120-Tetrahydrofuran	9.75	-0.73	-0.50
SI/sulf-RT-Toluene	2.66	3.02	10
SI/sulf -80-Toluene	9.2	2.85	9.65
SI/sulf -100-Toluene	11.05	-0.46	-0.06
SI/sulf -120-Toluene	12.77	-1.64	13.5

It should be noted that the skewness values represent the degree of bias of the roughness shape (asperity).

Skewness<0: Height distribution is skewed above the mean plane.
Skewness=0: Height distribution (peaks and pits) is symmetrical around the mean plane.
Skewness>0: Height distribution is skewed below the mean plane.

Also, it should be taken into consideration that kurtosis value is a measure of the sharpness of the roughness profile.

Kurtosis<3: Height distribution is skewed above the mean plane.
Kurtosis=3: Height distribution is normal. (Sharp portions and indented portions co-exist.)
Kurtosis>3: Height distribution is spiked.

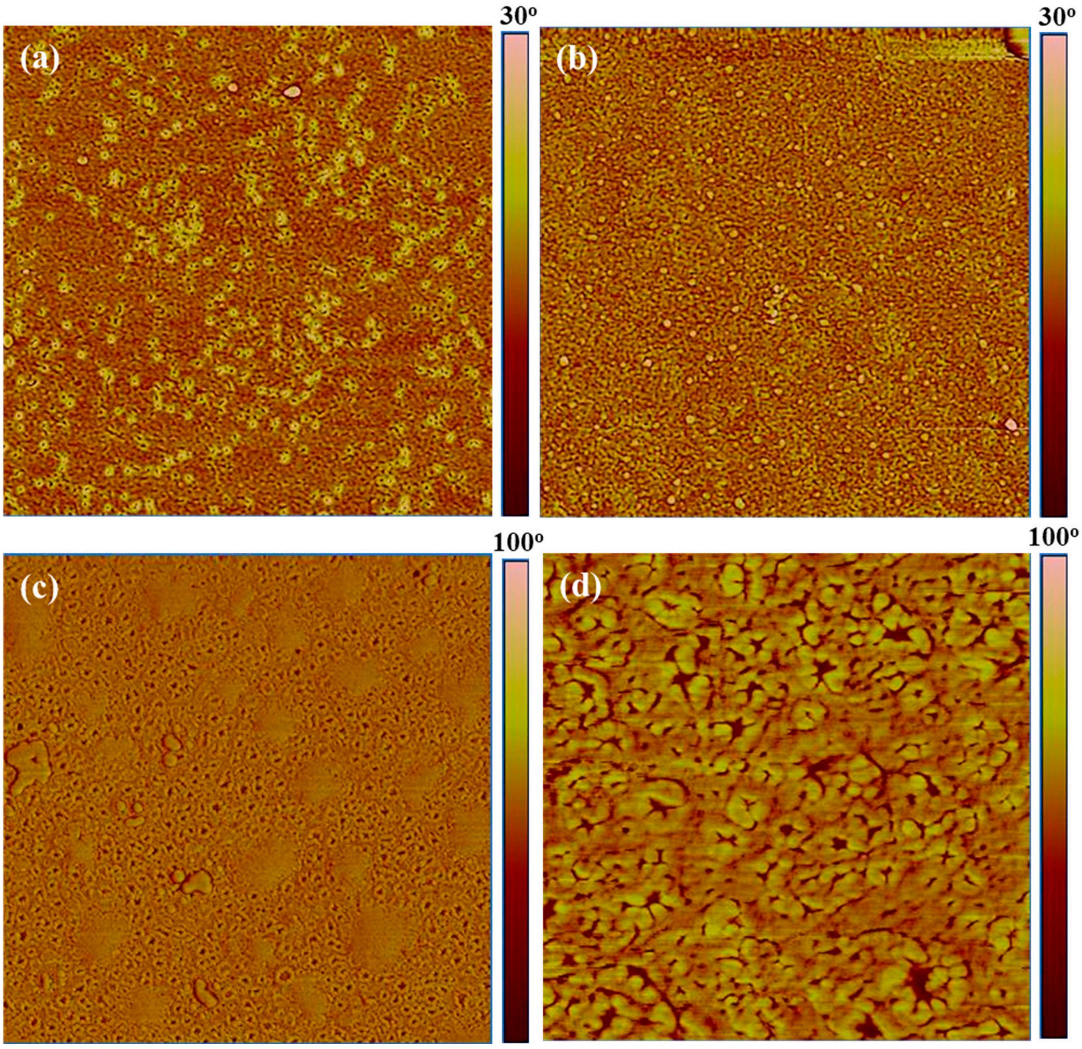

Figure S2: AFM phase images (3μm x 3μm except) corresponding to: the pristine initial diblock copolymer (SI) in (a) toluene/80°C, (b) cyclohexane/80°C and to the partially sulfonated (SI/sulf) copolymer (c,d) where possible micellar structures are illustrated in toluene/100°C (c) and toluene/120°C (d) respectively.

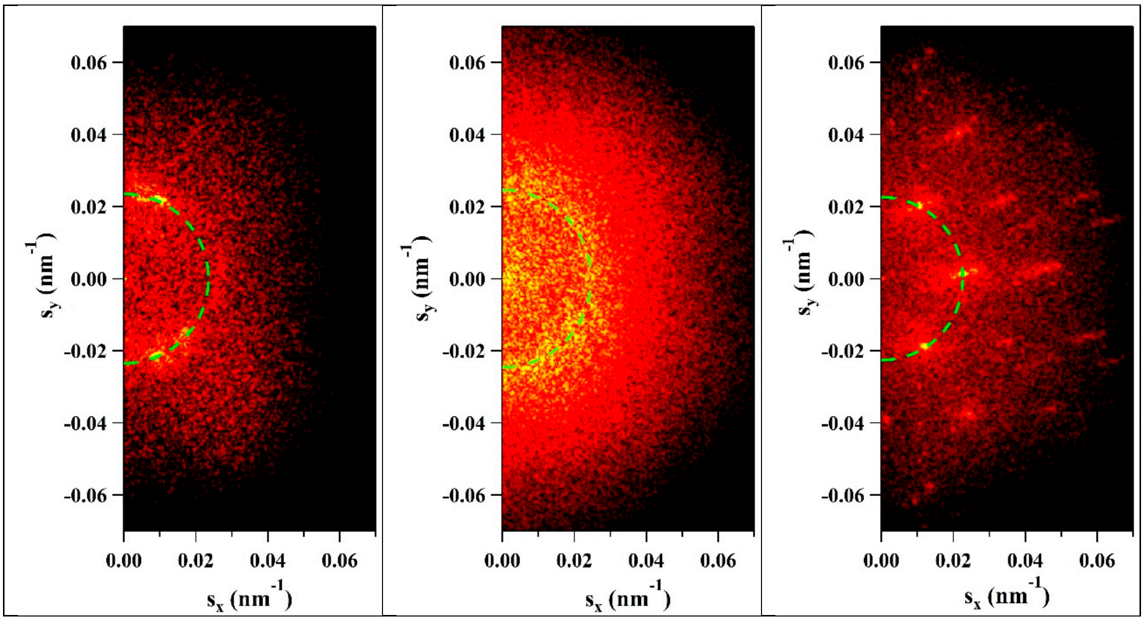

Figure S3: 2D Power Spectral Density functions corresponding to AFM images (Figs 4a,b and S2a) of the pristine initial diblock copolymer (SI) cast from toluene at R.T. (*left*), the same sample after annealing at 80 °C (*middle*) and the 100% hydrogenated (SEP) material in the as-prepared state (*right*). The dashed green circles are tranced through the fundamental diffraction peak.

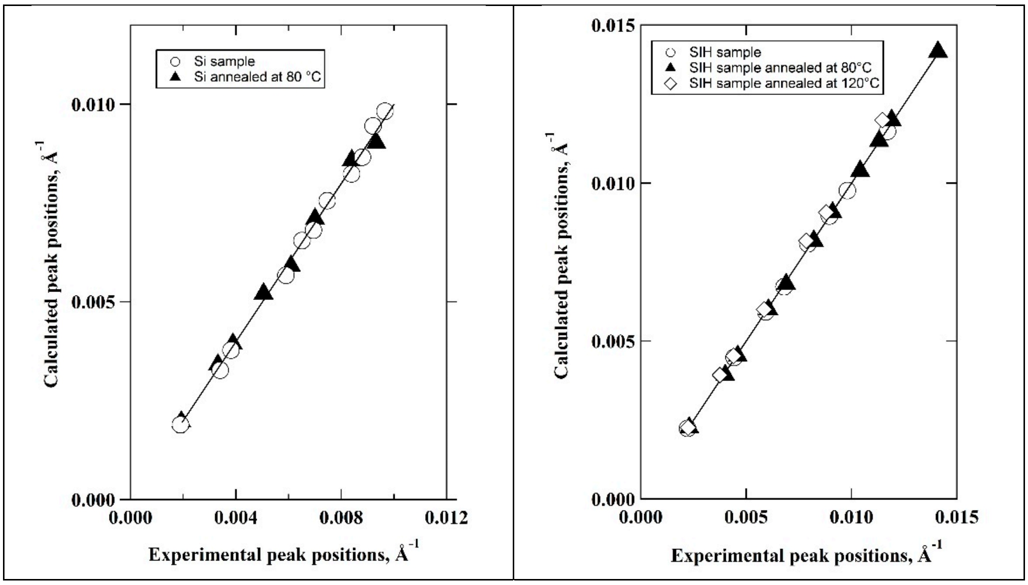

Figure S4: Comparison of the experimental and calculated d-spacings for samples SI cast from toluene at R.T. and upon annealing at 80 °C (left) and the SEP material cast from THF at R.T. and upon annealing at 80 °C and 120 °C (right). The solid lines correspond to equation y=x. (See main text for more details).

REFERENCES

1. van Krevelen, D.W.; te Nijenhuis, K. *Properties of Polymers: their correlation with chemical structure; their numerical estimation and prediction from additive group contribution*, 4th ed.; Elsevier: Amsterdam, Netherlands. **2009**; pp. 201-257.
2. Hansen, C.M. *Hansen Solubility Parameters a User´s Handbook*, 2th ed.; CRC Press, Taylor & Francis Group **2007**; pp. 1-17.
